# Biogas Production Systems and Upgrading Technologies: A Review

**DOI:** 10.17113/ftb.59.04.21.7300

**Published:** 2021-12

**Authors:** Martina Andlar, Halina Belskaya, Galina Morzak, Mirela Ivančić Šantek, Tonči Rezić, Vlatka Petravić Tominac, Božidar Šantek

**Affiliations:** 1 University of Zagreb, Faculty of Food Technology and Biotechnology, Pierottijeva 6, HR- 10000 Zagreb, Croatia; 2 Belarussian National Technical University, Mining Engineering and Engineering Ecology Faculty, Nezavisimosti Ave. 65, BY-220013 Minsk, Belarus

**Keywords:** anaerobic digestion, biogas production, different anaerobic bioreactor systems, biogas purification and upgrading technologies, digestate

## Abstract

The underutilized biomass and different organic waste streams are nowadays in the focus of research for renewable energy production due to the effusive use of fossil fuels and greenhouse gas emission. In addition, one of the major environmental problems is also a constant increase of the number of organic waste streams. In a lot of countries, sustainable waste management, including waste prevention and reduction, has become a priority as a means to reduce pollution and greenhouse gas emission. Application of biogas technology is one of the promising methods to provide solutions for both actual energy-related and environmental problems. This review aims to present conventional and novel biogas production systems, as well as purification and upgrading technologies, nowadays applicable on a large scale, with a special focus on the CO_2_ and H_2_S removal. It also gives an overview of feedstock and the parameters important for biogas production, together with digestate utilization and application of molecular biology in order to improve the biogas production.

## INTRODUCTION

**Fig. S1:**
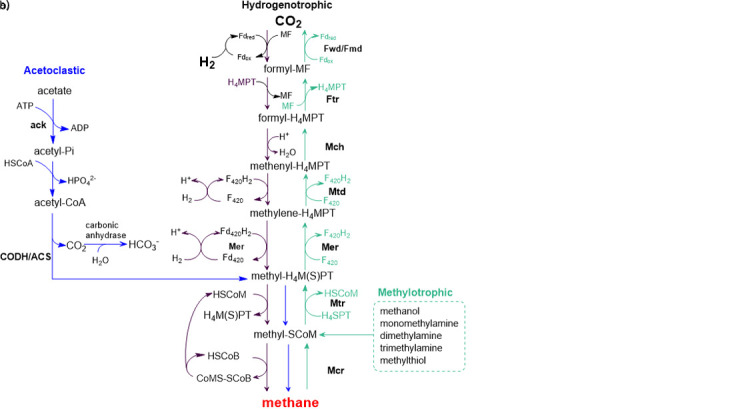
Microbial and metabolic characteristics of biogas production: a) the main phases of anaerobic digestion, and b) metabolic pathways of methane synthesis in *Methanosarcina barkeri* CM1. HSCoA=coenzyme A, HSCoB=coenzyme B, HSCoM=coenzyme M, MF=methanofuran, H_4_MPT=tetrahydromethanopterin, CODH/ACS=carbon monoxide dehydrogenase/acetyl-CoA synthase, ack=acetate kinase, Fwd/Fmd=formyl-methanofuran dehydrogenase/formyl-methanofuran-H_4_MPT formyl transferase, Fd=ferredoxin, Ftr=formyltransferase, Mch=methanopterin cyclohydrolase, Mtd=F_420_-dependent methenyle-H_4_MPT-dehydrogenase, Mer=methylene-H_4_MPT-reductase, Mtr=methyltransferase, Mcr=methyl-coenzyme M reductase (*3*)

Biogas is a renewable energy source that can be produced from different cheap recyclable organic waste streams combined with the reduction of greenhouse gas emission. Biogas production also contributes to economic benefits for farmers through the production of organic fertilizers, reduction of pathogenic microorganisms and removal of odours. Recent evaluations have indicated that the anaerobic digestion (AD) is an efficient alternative technology that combines biogas production with sustainable waste management (*1*, *2*). The AD is a bioprocess during which complex organic materials are decomposed in the absence of oxygen through the following phases: hydrolysis, acidogenesis, acetogenesis and methanogenesis (Fig. S1a (*3*)). The digestion of such complex organic biomass into a source of clean and renewable energy reduces the greenhouse effect and results in a production of two very valuable products: biogas and digestate. Biogas is composed of CH_4_, CO_2_, H_2_S, NH_3_, N_2_ and traces of water vapour (*4*). Digestate is the residue (decomposed organic feedstock) of biogas production that contains different macro- and micronutrients. It can be used as a soil fertilizer due to its good C/N ratio and homogeneity, nutrient availability and significantly reduced odour.

The individual biomass degradation steps are carried out by different consortia of microorganisms. In the first step of the AD, the complex organic material, rich in carbohydrates, proteins and fats, is hydrolysed by extracellular enzymes of hydrolytic bacteria to simpler compounds: mono- and oligomers, amino acids and fatty acids. These compounds are converted to short-chain fatty acids (SCFAs), alcohols, H_2_S, CO_2_ and H_2_ in an acidogenesis (fermentative) step. In the next step, acetogenesis, SCFAs and alcohols are oxidised into methanogenic substrates like acetic acid, H_2_, CO_2_ and water. The last step is methanogenesis in which products from acetogenesis are converted to CH_4_ and CO_2_. Methanogenesis can be conducted through the hydrogenotrophic, acetoclastic or methylotrophic pathways (Fig. S1b (*3*)). This is the rate-limiting step since members of *Archaea* domain are very sensitive to any changes in their environment. They can be easily influenced by many factors including the composition of the feedstock, feeding rate, volatile fatty acid (VFA) concentration or rapid changes in temperature and pH values, resulting in termination of methane production and process inhibition. The process conditions required for a stable one-stage AD system have to be as follows: pH=6.50-7.50, alkalinity as CaCO_3_ 1.5-4.0 g/L, volatile solids/total solids >45%, total ammonia nitrogen <1500 mg/L, C:N ratio 20-30 and C:N:P ratio 100-120:5:1 (*1*, *5*).

Low costs and the broad range of feedstock increase the biogas potential to be used for heat, steam, electricity or hydrogen production as well as a transportation fuel.

## RENEWABLE FEEDSTOCK FOR BIOGAS PRODUCTION

Biogas can be produced from various types of widely available organic feedstock such as animal manure and slurries, wastewater and sewage sludge, municipal solid waste, organic waste from dairy production and food industry, agricultural biomass, lignocellulosic residues (stalks, leaves, roots, seeds, seed shells), organic waste from households as well as energy crops. Industrial waste and agricultural biomass residues have been a promising feedstock without affecting natural resources, along with fewer risks and competition for food. As the expenses of lignocellulosic feedstock conversion to bioenergy exceed the price of fossil fuels, the development of economically viable production systems requires the use of low-value biomass and waste that are currently underutilized. Utilization of underused biomass feedstock is in alignment with the circular bio-economy concept by reducing the overall biogas production as well as waste treatment costs.

Depending on the used feedstock, the process of biogas production should be optimized in terms of composition of the substrate, total (TS) and volatile (VS) solids content, C/N ratio, organic loading rate (OLR), bioprocess parameters (temperature, pH, moisture content, loading rate, mixing intensity, retention time), presence of inhibitors (NH_3_, heavy metals, oil, grease, phenols, antibiotics, VFAs) and biodegradability (*6*). In the following subsections, an overview of lignocellulosic and non-lignocellulosic feedstock, energy crops and co-digestion substrates is presented.

Lignocellulosic feedstock comprises a complex and recalcitrant matrix, constituted of cellulose, hemicellulose and lignin, resistant to microbial hydrolysis (*7*). High crystallinity and degree of polymerization of cellulose, lignin as a physical barrier that limits the access of the enzymes, and cellulose interweaving by hemicellulose are the major barriers for lignocellulose application as a feedstock for anaerobic digestion. In addition, slow hydrolysis rate, long retention time, formation of toxic compounds and low methane yields have been observed when using lignocellulosic feedstock (*8*). To overcome these issues, with the intention to enhance degradation and consequently biogas production, the lignocellulose should be treated prior to the AD. Several pretreatment methods have been developed, including physical, chemical, thermochemical, oxidative and biological methods (*9*). Pretreatment is responsible for opening the structure of such a complex matrix, thereby reducing the cellulose crystallinity and making it more accessible, separate the components of the lignocellulosic biomass and remove the lignin. During the pretreatment, inhibitors such as furfural and hydroxymethylfurfural can be generated. Novel methods with ionic liquids, supercritical CO_2_ or oxidative pretreatment are promising techniques that avoid the use of strong acids/alkalis and the formation of inhibitory compounds (*10*).

The most utilized lignocellulosic feedstock for biogas production can be categorized into four main groups: (*i*) agricultural residues (*e.g.* bagasse, stalks of cotton plant and barley, empty fruit bunch from oil palm, different crop straws, corn cobs, maize and sorghum stover or peanut shell), (*ii*) fruit and vegetable waste, (*iii*) forestry residues (*e.g.* woody biomass like birch, eucalyptus, beech, cedar, pinewood or oak) and paper waste, and (*iv*) separated municipal solid waste (different organic materials, mainly from kitchen waste, from which lignocellulosic solid waste can be sorted out) (*11*). Table 1 (*12*-*27*) shows examples of lignocellulosic and non-lignocellulosic residues from agricultural and agro-industrial sectors, with the obtained biogas yields.

**Table 1:** Composition and biogas yield (*V*(CH_4_)/*m*(VS))/(m^3^/kg) from different lignocellulosic and non-lignocellulosic raw materials

Feedstock	*w*(TS)/%		*w*(VS)/%	C:N ratio	*Y*(biogas)/(m^3^/kg)		Reference
Lignocelllulosic material					
banana pseudostems		5	4	38:1.24	0.34	(*12*)	
forestry residues		75	64	325	0.21	(*13*)	
fruit waste		15-20	75	35	0.25-0.50	(*14*)	
garden waste		60-70	90	100:150	0.20-0.50	(*15*)	
grass		20-25	90	12:25	0.55	(*16*)	
grass silage		50	92	10:25	0.33	(*16*)	
maize silage		35	94	15-30:1	0.60-0.70	(*17*)	
palm oil fibre		76	78	44:2	0.37	(*18*, *19*)	
straw		70-90	80-90	80:100	0.15-0.35	(*20*)	
sugarcane bagasse		94	97	45:1.72	0.22	(*21*)	
wheat straw		98	93	58:1.34	0.27	(*20*, *22*)	
Non-lignocellulosic material							
cattle slurry		11	82	6-20	0.20-0.30	(*23*)	
food residues		20	92	50:2	0.15-0.39	(*20*)	
palm oil mill effluent		3.10	86	44:2	0.35	(*24*)	
pig slurry		7	86	3-10	0.25-0.50	(*23*)	
potato peel pulp		6-18	90	46:4	0.30-0.90	(*25*)	
slaughtering waste		15	80	4:1	0.30-0.70	(*26*)	
vinasse		1	90	12:1	0.24-0.30	(*27*)	

TS=total solids, VS=volatile solids

The main sources of biogas non-lignocellulosic raw materials are found in processing units of food, beverage, pharmaceutical and agro-industrial sectors (animal manure and slurries) together with a municipal solid waste. Animal manure is a highly favourable substrate for biogas production because it reduces CH_4_ and N_2_O emissions (*28*). However, due to low dry matter content (<10%), it is almost always mixed with lignocellulose residues to increase dry matter and the C/N ratio, making the bioprocess more efficient and economically profitable (*29*, *30*). The chicken manure is problematic, not only because of wood chip fractions used as bedding material but also because of high nitrogen, uric acid and protein concentrations, which is in correlation with NH_3_ concentration and process inhibition (*31*). The composition of different non-lignocellulosic residues generated by urban food processing and agro-industrial sectors is shown in Table 1.

Energy crops are cultivated feedstock (grains, silage and grasses) composed of cellulose, hemicellulose, lignin and monosaccharides like glucose, fructose and sucrose, fructans, extractives and pectins (*32*). They are primarily cultivated for biogas production and can be grown on soils that are not intended or appropriate for food production. They can be digested either alone or in co-digestion with other materials. Energy crops should comply with the following characteristics: high production yield (dry matter per ha), high methane yield ((*V*(CH_4_)/*m*(VS))/(m^3^/kg)), low energy input for the cultivation of the plant, harvesting, and processing; low expenses; low content of contaminants; cultivation without using pesticides, herbicides and fertilizers; low nutrient demand; and lastly short growing season (*33*). Still, the production, harvesting time, nutrient composition, conservation and pretreatment technologies have to be improved for each. They can be divided into four categories: (*i*) sugar-based crops (sugar cane and beet, sweet sorghum), (*ii*) starch-based crops (wheat and corn), (*iii*) lignocellulose-based crops (fodder grass and switch grass), and (*iv*) woody (miscanthus, willow, poplar and oak). Grains, maize and grass are the most commonly cultivated energy crops with a high net energy yield per hectare (*34*). Recent studies on the production of biogas from energy crops point out dedicated energy crops specifically grown for energy and fuel production. These include corn and sugar cane, non-food crops as poplar trees and switchgrass (*35*). Biogas and crop yields with the calculated energy potential of diverse energy crops are listed in Table 2 (*15*, *34*).

**Table 2:** Yields of crop, biogas (*V*(CH_4_)/*m*(VS))/(m^3^/kg) and energy obtained from different energy crops (*15*, *34*)

Feedstock	*Y*(crop)/(t/ha)	*Y*(biogas)/ (m^3^/kg)	*Y*(energy)/(GJ/ha)
alfalfa	7.50-16.50	340-500	82-266
barley	3.60-4.10	353-658	41-87
flax	5.50-12.50	212	38-85
Jerusalem artichoke	9-16	300-370	87-191
kale*	240-334	6-45	46-484
leaves of sugar beet*	9.20-18.40	0.40-0.80	70-226
*Miscanthus*	8-25	179-218	46-176
oats (grain)	4.10-12.40	283-492	33-146
oilseed rape	2.50-7.80	240-340	19-85
rhubarb	2-4	320-490	21-63
sugar beet*	9.20-18.40	0.40-1	70-226
sunflower	6-8	154-400	30-103
triticale	3.30-11.90	337-555	36-213
wheat (grain)	3.60-11.75	384-426	45-161

*Y(biogas)=(*V*(CH_4_)/*m*(dry matter))/(m^3^/kg), VS=volatile solid

Simultaneous conversion of two or more substrates is necessary to improve the yield of anaerobic digestion, C/N ratio, bioreactor stability and obtain a well-balanced content of nutrients and buffer capacity. Therefore, animal manure is often mixed with carbon-rich substrates such as lignocellulose to provide a better nutritional balance in the AD system (*36*). Advantages of co-digestion include: (*i*) increased biogas and methane production compared to single substrates treated individually (*37*), (*ii*) better stability of digestion and homogenization (*38*), (*iii*) avoiding the use of additional fertilizers (*39*), (*iv*) greenhouse gas emission and odour reduction (*38*), (*v*) dilution of potentially toxic chemicals like ammonia and thus improving the stability of digestion (*40*), and (*vi*) avoiding the inhibition of the AD caused by the VFAs accumulation and a decrease in pH value (*41*).

## IMPACT OF DIFFERENT BIOPROCESS PARAMETERS ON THE BIOGAS PRODUCTION

The performance and efficiency of the AD, that is, the growth and activity of anaerobic microorganisms, especially methanogens, has been affected by some notable parameters monitored during the bioprocess. These parameters include temperature, pH, volatile fatty acids (VFAs), total (TS) and volatile solid (VS) concentration, organic loading rate (OLR), hydraulic retention time (HRT), solid retention time (SRT), toxic compounds, C/N ratio, CO_2_ pressure, mixing and shear stress. It is essential to maintain these parameters in a range optimal for microbial growth and activity in order to decrease the risks of process failure (*42*).

The AD can be performed under three different temperature conditions: psychrophilic (<25 °C), mesophilic (25-55 °C) and thermophilic (55-70 °C). Under psychrophilic conditions, the conversion efficiency of VS to biogas is lower than under the other temperature regimes. Low temperatures correlate with VFA accumulation, a slightly higher fouling rate and significant methane loss (*10*). On the other hand, under thermophilic conditions the shortest HRT, higher microbial metabolic rates, degradation rates of organic matter, and the highest organic loading capacity have been established. Despite that, the high temperature raises NH_3_ concentration, resulting in unstable AD and inhibition. In addition, some equipment (pumps, gasholder and mixers) can be destroyed by high temperatures (*1*). The mesophilic process does not require any specific care related to the selection of materials. It generally runs smoothly and requires little thermal energy to maintain the stable bioprocess temperature.

The direct relation between the process temperature and the HRT is noticeable. The minimum retention time needed for the psychrophilic stage is 70 to 80 days, for mesophilic 30 to 40 days and for thermophilic 15 to 20 days (*43*). Temperature variations have negative impact on the microbial consortium involved in the AD process. In the plant design process, it is crucial to prevent rapid temperature changes during plant operation. Temperature changes in bioreactors have to be slow so that the existing microbial populations have enough time to adapt to the new environmental conditions without affecting the efficiency and biogas production.

Optimal growth of different microbial groups involved in the AD happens under different pH values. This is the crucial reason for the separation of acidogenesis and methanogenesis. Methane production takes place within a relatively narrow pH interval, from about 6.5 to 8.5, while acidogenesis from 5.0 to 6.0 (*44*). As for the temperature, drastic changes in pH values inhibit the AD process. The solubility of CO_2_ in water and the formation of carbonic acid is lower at higher temperature, which means that the pH value in thermophilic bioreactors is higher than in mesophilic ones. The pH value can be controlled by adding the NaOH and NH_3_ or through the bicarbonate buffer system. The excess of Na^+^ ions, concentrations >3.5 g/L, leads to bioprocess inhibition (*45*). In addition, the pH value inside the bioreactor depends on the partial pressure of CO_2_ and on the alkaline and acid concentrations in the liquid phase.

In microbial cells, CO_2_ can participate in the metabolism as an intermediate, carbon donor, electron acceptor or final product of metabolic reactions. It can also contribute to the medium buffer system through the carbonate equilibrium. The response of microbial cells to the CO_2_ partial pressure in the environment depends on its concentration. The high CO_2_ partial pressures (4-30 MPa at 20-50 °C) used for sterilization are related to the cytoplasm acidification, inactivation of different enzymes and cell disruption. The lower CO_2_ partial pressure (0.01-1 MPa) is usually related to the decreased intracellular pH and therefore reduced microbial activity of denitrifying bacteria when dissolved CO_2_ concentrations were up to 30 g/L (*46*). Under these conditions, elevated CO_2_ partial pressure can be related to the direct inhibition of carbon metabolism, enzyme activities and substrate consumption in order to increase buffer substance concentrations and to prevent pH drop (*47*, *48*). The addition of CO_2_ at atmospheric pressure in AD bioreactors coupled with stoichiometric H_2_ supply is related to the increased CH_4_ production due to enhanced hydrogenotrophic methanogenesis (*49*). CO_2_ can be indirectly converted into CH_4_ through homoacetogenesis affiliated with acetoclastic methanogenesis. This approach was used to explain the enlarged CH_4_ production after direct CO_2_ injection in pilot-scale anaerobic food waste digestion and two-stage anaerobic sewage digestion (*50*). Higher CO_2_ partial pressure is also connected to the changes in microbial population diversity, activity, structure and interactions (*51*). Furthermore, it also has an impact on the propionate and butyrate accumulation that are correlated with AD disturbance through the pH changes, increased OLR and non-harmonized acidogenesis and methanogenesis (*46*).

VFAs are intermediate compounds (acetate, propionate, butyrate, lactate) produced during the hydrolysis and acidogenesis stages of the AD process. A reliable factor for the AD stability estimation is the VFA concentration, which is determined by their production and consumption rates, presence of inhibitors, temperature instability, loading rate and feedstock characteristics. Accumulation of these acids, more than 1.5-2 g/L, can notably reduce the pH and afterwards inhibit the methanogenesis (*52*). Co-digestion with other raw materials or using a two-stage digestion system are suggested to prevent the inhibition of biogas production by VFA accumulation (*53*). Different analytical methods have been performed to measure VFA concentration, like titration, photometric, colorimetric and chromatographic methods, steam distillation, alkalinity or buffering capacity measured in mg/L of CaCO_3_, infrared spectrometry, high-pressure liquid chromatography (HPLC) or gas chromatography (GC).

The toxic compounds can be brought into the bioreactor together with the feedstock (oil, grease, phenols, antibiotics, heavy metals and volatile aromatics) or generated during the process (NH_3_, H_2_S, long chain fatty acids and VFAs) (*54*). The toxicity level of some inhibitors depends on their concentration and pH value in the bioreactor. The NH_3_ is considered toxic at pH>7.0, while H_2_S shows its inhibitory effects at pH<7.0 (*55*). The temperature influences the equilibrium between the NH_4_^+^ ions and free NH_3_. Therefore, NH_3_ inhibition can be more pronounced under thermophilic than mesophilic conditions due to enlarged concentrations of free ammonia nitrogen (FAN) at higher temperatures (*56*).

The C/N ratio should be continuously controlled and monitored to achieve a high degradation rate of organic materials and consequently bioprocess potential. The C/N ratio is correlated with the nutrient composition of the substrates. An example of a balanced C/N ratio is co-digestion of animal manure that has a low C/N ratio and carbon-rich substrates such as silage (*36*). Above- or under-optimal C/N ratio is related to the unfavourable impact on the rate of methane production and activity of microbial culture (*1*). The optimal C/N ratio for the AD is in the range between 20 and 30 (*57*). Besides C/N ratio, phosphorus and sulphur are also essential to ensure maximal growth and activity of the microorganisms involved in the AD. The optimal ratio of these macronutrients is suggested to be C/N/P/S=600:15:5:1 (*58*).

Total solids (TS) represent the total content of organic and inorganic compounds in the substrate while volatile solids (VS) represent the content of the organic compounds:



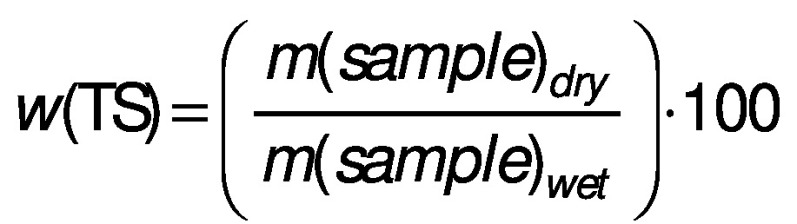





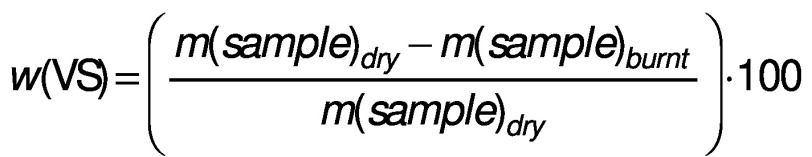



The TS and VS mass fractions (in %) of the substrates give useful information about the biogas yield that is potentially produced. Moreover, the efficiency of mechanical parts such as pipes, cutters, pumps and mixers, selection of bioreactor as well as overall AD process relies on the TS mass fraction. In other words, the TS of the substrate organic matter and TS during the process are the two main factors for selecting the type of system and bioreactor design (*59*).

Organic loading rate (OLR) is the operational parameter that indicates how much organic dry matter (kg) can be introduced in the bioreactor per working volume *V* (m^3^) and time *t* (day) (*60*) as per the following equation:



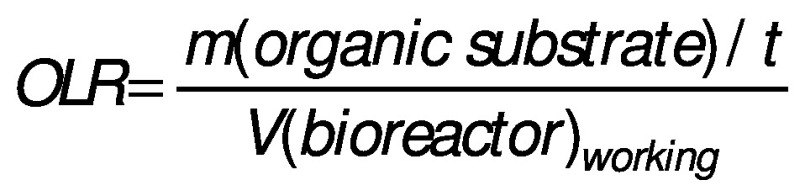



The OLR depends on the type of the substrate and bioreactor and should be increased slowly by starting at the OLR as low as 0.5 kg/m^3^/day and by giving enough time to the microorganisms involved in the AD process to adjust (*61*). An appropriate OLR in the continuous stirred tank reactors (CSTR) with mesophilic conditions varies between 3 and 5 kg/m^3^/day depending on the type of the substrate (*62*). Excessively high OLRs boost hydrolysis and acidogenesis phase performance, leading to high VFA production, acidification and inhibition of methanogenesis (*42*). Higher OLR is preferred from the technical point of view since smaller bioreactor would be required. In this case, the above-mentioned limitations related to high OLRs can be overcome by using two-stage anaerobic processes (*61*).

Hydraulic retention time (HRT) is an average period of time (*t*/day) at which the substrate stays inside the bioreactor and solid retention time (SRT) is a retention time of solids in the bioreactor:



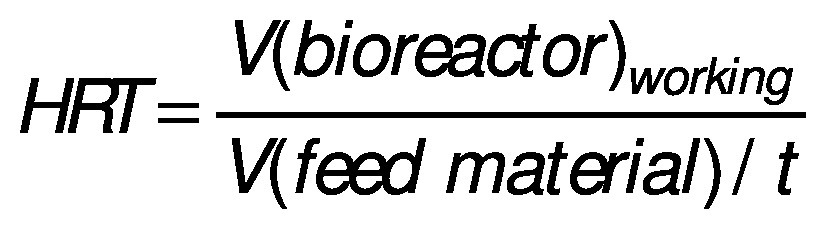





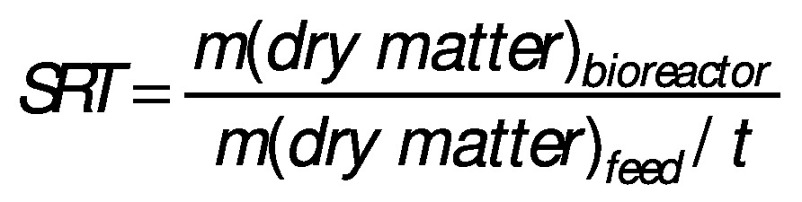



Since the HRT regulates the rate of bioconversion, it has to be long enough to avoid biomass washout. In other words, the number of microorganisms removed with the effluent should not be higher than the number of produced microorganisms per litre. The main parameters for adjusting HRT and SRT that have to be considered are the substrate composition and the AD temperature. The HRT and SRT are correlated with the economic aspects of biogas plants, meaning shorter retention times are associated with lower bioreactor volume as well as with lower capital and maintenance costs (*63*, *64*). However, from a microbial point of view, shorter time can decrease substrate affinity, increase the risk of biomass washout, and lower CH_4_ yields (*44*). Retention time values can range from a few hours up to 30 days, as reported by Jeong *et al.* (*63*). The optimal value of HRT and SRT should be determined from case to case by considering feed characteristics, mixing, sludge properties, bioreactor design and configuration (*65*).

## BIOGAS PRODUCTION SYSTEM CONFIGURATIONS

Anaerobic digestion is carried out in a hermetic tank called digester or anaerobic bioreactor where organic waste is mixed with microbial culture and converted to biogas. There are numerous types of anaerobic bioreactors made of reinforced concrete, carbon and stainless steel, or specially coated steel, brick and plastic. They can be constructed in the shape of silos, troughs, basins or ponds. Their configuration and size depend on the scale of biogas plant (small household installations, large commercial plants), solid content of the feedstock (wet digestion – less than 15% and dry digestion - from 16 to 40% (*m/m*) on dry matter basis), temperature of digestion (psychrophilic, mesophilic, thermophilic), cultivation manner (batch, continuous), and number of stages (single-stage, two-stage, multistage).

**Fig. 1:**
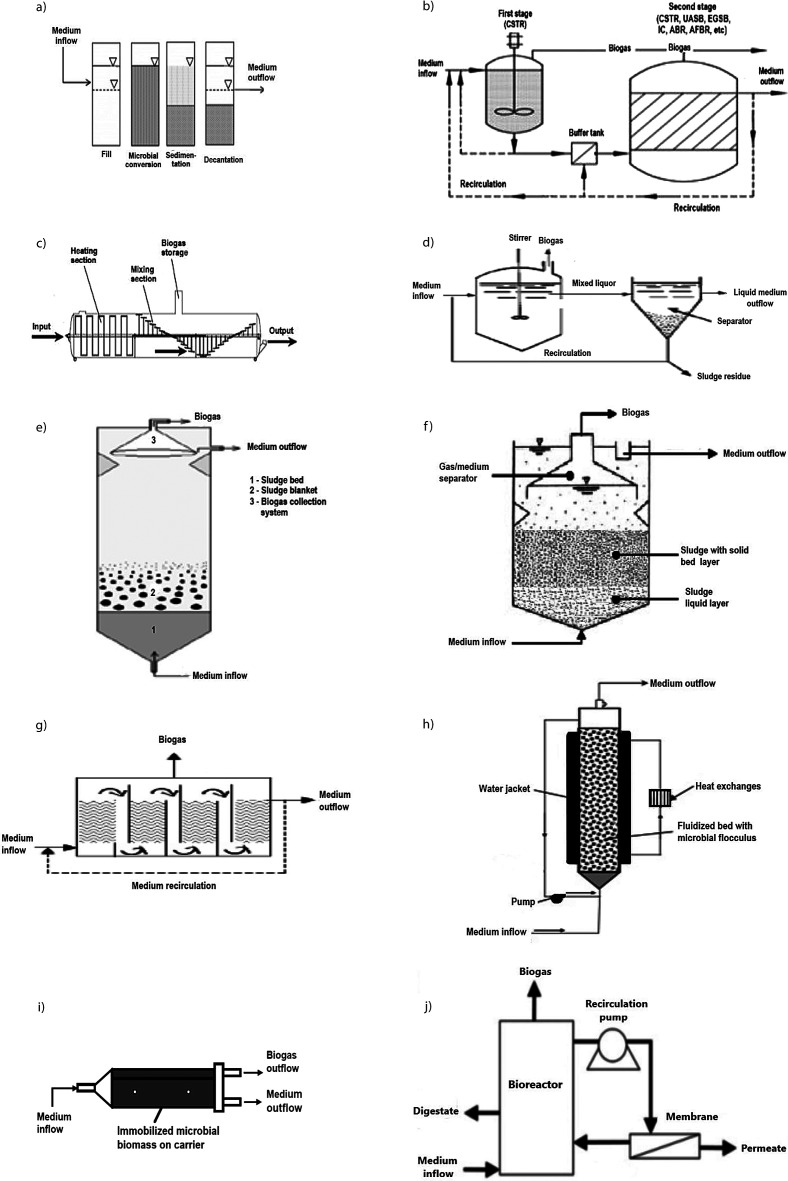
Different bioreactors for anaerobic digestion: a) anaerobic sequencing batch reactor (ASBR), b) continuous stirred tank reactor (CSTR) in a two-stage system, c) anaerobic plug-flow reactor (APFR), d) bioreactor with sludge retention system, e) up-flow anaerobic sludge blanket (UASB) bioreactor, f) up-flow anaerobic solid-state (UASS) bioreactor, g) anaerobic baffled reactor (ABR), h) anaerobic fluidized bed reactor (AFBR), i) horizontal-flow anaerobic immobilized biomass (HAIB) bioreactor, and j) anaerobic membrane bioreactor

The fundamental requirements of bioreactor construction design are to shorten the start-up period, reduce washout of active biomass, be easily adapted for variations in feedstock content, ensure process stability and produce a high volume of biogas. The choice for the type of bioreactor has to take into account some aspects including cost of construction, operation and energy consumption, abilities for effluent and digestate disposal, climate conditions, infrastructural support and waste type and composition. Biogas production recovers energy from organic waste as well as significantly alleviates the impact of the waste on the environment. Cost of installation, operation and maintenance are the main parameters that considerably affect the biogas economics. Many different bioreactor configurations can be used for biogas production and generally they are classified in three main groups: (*i*) conventional anaerobic bioreactors, (*ii*) sludge retention bioreactors, and (*iii*) anaerobic membrane bioreactors (Fig. 1).

### Conventional anaerobic bioreactors

#### Anaerobic sequencing batch reactor

Anaerobic sequencing batch reactor (ASBR) operates in the following cyclic stages: substrate feeding, microbial conversion (biogas production), sludge settling and substrate discharge (Fig. 1a). ASBR ensures conditions for microbial AD from substrate consumption to biogas production. Microbial cultures in an ASBR are exposed to the changes of substrate concentration over cycle period and therefore higher rates of substrate conversion coupled with efficient biomass separation are observed. In ASBR, high substrate conversion rates are observed at the beginning of the cycle although relatively high substrate concentrations are present. At the end of a cycle, substrate concentration is reduced and consequently biogas production is also diminished. Under these new circumstances, sludge settling conditions are established. The ASBR serial batch operation mode enables that hydraulic residence time is independent of the solid residence time due to the fact that vessels have decanter function whenever the stirrer is turned off. The ASBR is relatively easy to operate, it is simply constructed, with low input process and mechanical requirements, cost-effective but only applicable for smaller volumes (*66*). Wide variations in wastewater strength, under mesophilic and thermophilic conditions, are appropriate for treatment in the ASBR. However, channelling and clogging, poor contact of the substrate with microorganisms, and small capacity are some of the ASBR limitations (*1*).

### Continuous stirred tank reactor and its modifications

The most commonly used reactor configuration is the CSTR (Fig. 1b), mostly used in wet digestion systems for raw materials with higher TS mass fractions and slurries (animal manure, lignocellulose feedstock, organic industrial wastes). The CSTR can operate under different temperatures and OLRs (2-5 kg/m^3^/day). The bioreactor consists of a tank with one or more mechanical stirrers, which can work continuously or periodically. The stirring can be achieved by mechanical agitation (stirrers), hydraulic (pumps) or by pneumatic mixing (biogas recirculation). The mixing is a key factor that enables broth homogeneity, better contact of microorganisms with the substrate, and constant temperature in the whole bioreactor. It prevents the formation of microbial floccules and accumulation of VFAs that cause inhibition of biogas production (*67*). Since the suspended biomass in the medium is removed together with the slurry, the HRT and SRT have to be equal to avoid biomass washout. The main disadvantages of this system are long retention time, mixing energy consumption and difficulties in retaining a high microorganism concentration (*68*). Demirel *et al.* (*69*) found that the two-phase or series of CSTRs can improve biomass conversion efficiency and biogas yield.

Temperature-phased anaerobic digestion (TPAD) system consists of two serially connected CSTRs. The first bioreactor of TPAD system is characterised by higher temperature (>50 °C, and residence time 2-4 days) and the second bioreactor by moderate temperature (~35 °C, and residence time 12-20 days). In the first bioreactor, conditions for organic substrate hydrolysis are established in order to improve substrate digestibility. In the second bioreactor of the TPAD system methane is produced. The obtained digestate can be reused in agriculture (*70*).

Pump-mixed anaerobic digestion (PMAD) system can be designed as a one- or two-stage system. The one-stage PMAD takes place in only one bioreactor vessel. The first bioreactor of the two-stage PMAD system is usually standard stirred tank bioreactor where substrate hydrolysis takes place. The second bioreactor is pump-mixed (circulation) bioreactor where methanogenesis occurs under increased pressure in order to achieve higher CO_2_ solubility in the medium. Therefore, relatively high CH_4_ content (~65%) can be observed in the biogas outlet. It is known that medium recirculation by a pump has a strong impact on the flow pattern in the bioreactor and consequently higher CH_4_ quantity is released from the medium due to its poor solubility (*71*-*73*).

High-pressure anaerobic digestion (HPAD) is usually constructed as a two-stage system. In the first bioreactor of the HPAD system (usually CSTR), substrate hydrolysis occurs and in the second biogas production together with *in situ* upgrading by using high pressure (CSTR or column bioreactor) (*74*, *75*). In the second bioreactor of the HPAD system it is possible to obtain over 90% CH_4_ in the biogas outflow. Therefore, this biogas can be directly used in local grids or industrial processes. The large difference in solubility between CH_4_ and CO_2_ is a driving force of HPAD system. This effect is the most obvious at higher pressures in the bioreactor with a pressure valve for biogas release. Under the increased pressure, the CH_4_ content increases in the bioreactor headspace while the concentrations of CO_2_ and other gasses (*e.g.* H_2_S) grow in the liquid medium phase. The impact of increased medium CO_2_ concentration on the HPAD system performance has been studied only through the changes of medium acidity. Until now, the impact of increased medium CO_2_ concentrations on the microbial metabolism during anaerobic digestion under the conditions of high pressure have not attracted research attention (*46*).

### Anaerobic plug-flow reactor

The anaerobic plug-flow reactor (APFR) is a long, vertical or horizontal tank (Fig. 1c), generally more efficient in converting a substrate to biogas and offers more stable operation than the CSTR. A high amount of microbial sludge accumulates along the length of the bioreactor. Because of the bioreactor shape, the feedstock circulates slowly from the front side to the discharge side, forming a plug-flow regime through the bioreactor. It is usually designed without a mixer but some can have internal baffles or mechanical mixers. The construction of the APFR is relatively cost-effective and they are usually used in dry digestion to treat substrates with high TS mass fraction (11-14%) (*25*).

### Bioreactors with sludge retention systems

Until now, many anaerobic bioreactors have been designed, operated, studied and improved in their construction and efficiency. Methanogenesis, as the rate-limiting step, and slow-growing members of the domain *Archaea*, have been the most important challenges in biogas production. To maintain active members of the domain *Archaea*, enhanced anaerobic high-rate bioreactor systems, such as bioreactors with sludge retention system, have been developed, which was an important milestone for anaerobic digestion technology (*76*). Bioreactors with sludge retention systems were constructed to operate at a short HRT and long SRT with the purpose of maintaining high concentration and activity of microorganisms, improving the sludge stabilization and increasing the loading capacity of the system. They have been modified in the sense to boost the rate of organic waste degradation, reduce retention time and increase organic waste loading and biogas production (*76*). Bioreactors with attached microorganisms can be constructed as sludge blanket, expanded-, fluidized- and fixed-beds.

### Anaerobic contact reactor

The anaerobic contact reactor (ACR) is a mechanically stirred vessel (Fig. 1d) with a solid-liquid separator (gravity sedimentation tank, lamella clarifier or sludge flotation unit) for recycling of microorganisms. Because of the mechanical stirrer, the content of the ACR bioreactor is well mixed. The effluent from the tank flows into the separator while the settled solids are recycled back into the bioreactor. The ACRs are capable of treating a wide range of industrial effluents like the pulp and food industry wastewaters, paper mills and waste with high TS mass fractions. The HRT is short; oscillations in organic loading are well tolerated. The ACR bioreactor can handle OLR expressed as total oxygen demand (COD) up to 8 kg/(m^3^/day) with COD removal efficiency of 85-95% (*77*).

### Up-flow anaerobic sludge blanket bioreactor

The up-flow anaerobic sludge blanket (UASB) bioreactor is a cylindrical or rectangular column with a gas separator on the top (Fig. 1e). At the bottom of the bioreactor, a dense blanket of flocculated sludge is formed through self-immobilization of microorganisms. The combined action of the gravity and the substrate upward flow suspends the sludge blanket in the bioreactor and therefore effluent recycling is not necessary. Change in the substrate type and process conditions impacts the sludge quality and composition of the microbial community (*78*, *79*). The main features and benefits of the UASB bioreactors are compactness, high loading rates, low sludge production, short HRT and SRT times, and low operational cost with high methane production rates. The UASB bioreactor belongs to high-rate system bioreactors, which are typically run at HRTs less than 5 days and are used for wastewater treatment. Substrates with high mass fractions of TS are not appropriate to be treated in this type of bioreactor due to the tendency of substrate particle accumulation (*76*).

Mixing, caused by the up-flow of feedstock and by the rising gas bubbles, is insufficient, resulting in creating dead zones. In addition, the beginning of the anaerobic digestion can be prolonged because of the slow formation of granular sludge, which depends on operational conditions (pH, temperature, HRT) and used substrates (organic matter content). These drawbacks can be overcome by using a two-stage digestion system (*1*, *76*) or UASB bioreactors with membrane-based retention of sludge granules (*80*). Despite that, UASB bioreactors have been used for wastewater treatment due to simplicity, robustness and high efficiency (*81*).

The composition of the microbial community in the UASB bioreactor was studied at different substrates (ethanol, glucose) and temperatures (37, 45, 50 °C) by Li *et al.* (*78*). The dominant methanogens in the UASB bioreactor were from genera *Methanosaeta, Methanobacterium, Methanosarcina* and *Methanomassiliicoccus*. Increasing the temperature to 50 °C, the number of *Methanobacterium* decreased, while that of *Methanosaeta* increased. When ethanol was used as a substrate, the number of *Methanosaeta* was higher than with the glucose. In the study of Lu *et al*. (*79*) different ratios of organic materials and sulfate (COD/SO_4_^2−^) in the UASB bioreactor were investigated. It was observed that *Syntrophobacterales* were substituted with *Desulfovibrio* and by decreasing the COD/SO_4_^2−^ ratio, the composition of microbial consortium was changed.

### Expanded granular sludge bed bioreactor

The expanded granular sludge bed (EGSB) bioreactor is a modified version of the UASB with a large height to diameter ratio (10:1 up to 25:1) and a broader top cross section. The high height/diameter ratio allows higher up-flow velocities, from 6 up to 30 m/h for liquid and 7 m/h for gases. The lower section, containing the suspended granular sludge, is tall and narrow, resulting in an expanded sludge bed, better substrate-biomass contact, higher throughput, and improved internal mixing without dead zones (*1*, *23*, *82*). The upper section is a settling zone (gas-liquid-solid separator zone) which allows separating the treated wastewater from the granular sludge and gas. As a result of the high velocities, the medium is more expanded and thus the substrate-biomass contact is better, which allows it to work at high OLR values expressed as COD (40 kg/(m^3^/day)) and low HRT (0.2-2 days) (*82*). If the mixing of the treated effluent is not sufficient, the effluent can be recirculated by the pump to the bioreactor bottom to increase the flow rate.

Compared to the UASB, the EGSB bioreactor has higher permeability, smaller ecological footprint, and can be used for medium- and low-strength wastewater treatment containing soluble organics and lipids (*18*). The EGSB bioreactors have been applied to treat many kinds of wastewaters: brewery, starch, commercial laundry, domestic, municipal and pharmaceutical wastewater (*82*-*84*), but it is not adequate for the removal of suspended solids and colloidal organic matter due to the high velocity of the liquid up-flow. Additionally, problems with biomass retention result in granule disintegration and wash-out or the appearance of fluffy granules. It is suitable to work under psychrophilic conditions, even at a temperature as low as 10 °C. Many authors have studied hydrodynamics, kinetics, different inhibition effects, and start-up and operation characteristics in the EGSB bioreactors (*82*, *83*, *85*).

### Up-flow anaerobic solid-state bioreactor

The up-flow anaerobic solid-state (UASS) bioreactor is a novel design of bioreactor intended to treat lignocellulosic biomass (corn silage and barley straw) and organic solid wastes. The configuration of the UASS bioreactor is separated into three sections, liquid section at the bottom, solid-state bed in the middle and top liquid section with a sieve that works as a three-phase separator (Fig. 1f). The solids are introduced through the bottom of a column. In order to prevent VFA accumulation, the process liquids are recirculated through anaerobic filters. Recirculation of the microbial biomass is achieved by pumping it to the bioreactor bottom and then returning it in the bioreactor. The study of Mumme *et al.* (*17*) showed that the UASS bioreactor hydrolytic and methanogenic performance is among the highest reported for the digestion of solid biomass. Maize silage and barley straw have been used as a feedstock. The increase of VS loading rate from 7.1 to 17 g/(L·day) resulted in the overall methane yield decrease from 0.384 to 0.312 L/g. The contribution of the anaerobic filters to the methane yield was increased from 12 to 70%. The chemical oxygen demand (COD) of VS also decreased for 86-93% with a maximum hydrolysis rate of 16.4 g/(L·day). The authors concluded that the UASS bioreactor is a promising alternative for AD of different organic wastes. Likewise, Pohl *et al.* (*86*) demonstrated that it is feasible to digest straw in the UASS bioreactor. The UASS bioreactor was able to handle the fermentation of lignocellulosic waste expressed as VS up to 6 g/(L·day) as a single-stage system under thermophilic conditions.

### Anaerobic baffled reactor

The anaerobic baffled reactor (ABR) is a multi-section bioreactor assembled in a form of an elongated series of vessels separated by baffles that fully or partly divide the bioreactor in compartments (Fig. 1g). The baffles direct the flow of the liquid generating a plug-flow regime. Anaerobic sludge slowly rises and settles in each compartment, resulting in a long cell retention time (~100 days) and long HRT (~20 h) (*87*). The ABR is very effective for the treatment of high-strength effluents, it has long biomass retention times and can overcome the risk of clogging and sludge bed expansion. Besides that, the ABR has several disadvantages: treating high-strength effluents favours the development of a loading shock in the first compartments, slow growth rates of methanogens, long start-up process, and the possibility of the sludge washout at high hydraulic stresses. These disadvantages can be prevented by the baffle modification or by re-designing the compartments. The baffles can be placed at a certain angle with the peaks facing each other, which leads to a constant flow rate change and allows better contact between the microorganisms and the substrates. Furthermore, the compartments were redesigned in regards to volume ratio according to the different retention times of bacteria to create favourable environmental conditions for both acidogens and methanogens (*88*). The compartments might serve different purposes for enhanced production of biogas. For instance, Ran *et al.* (*89*) studied simultaneous production of biogas and hydrogen from organic waste in a four-compartment ABR. The first compartment was designed for hydrogen production while others were for methane production.

### Internal circulation bioreactor

The internal circulation (IC) bioreactor is structurally derived from the serial connection of two UASB bioreactors. The IC bioreactor has a large height to diameter ratio (8:4), which allows high organic loading rates expressed as COD (20-50 kg/(m^3^·day), retaining a good granular sludge activity, steady operation, resistance to shock loading and high bioprocess efficiency (*90*).

The wastewater is introduced at the bottom of the bioreactor in the main treatment zone with the most part of granular sludge. In the intermediate gas-liquid-solid zone the rising biogas is separated from the liquid and collected in a riser. Beyond the intermediate gas-liquid-solid zone is a polishing zone with a fine granular sludge where residual organics are removed. The biogas is further purified and collected in the upper gas-liquid-solid zone. At the top of the bioreactor, biogas passes through the gas-liquid separator and leaves the bioreactor. In the IC bioreactor, the efficient formation of granular sludge plays an important role and has been influenced by wastewater characteristics and operational control. The external and internal recirculation system of the liquid ensures suitable hydrodynamic shear force in the IC bioreactor and promotes sludge granulation and biomass retention.

### Anaerobic fluidized bed reactor

The anaerobic fluidized bed reactor (AFBR) is characterized by a two-phase mixture of fluid and small inert particles (fine sand, aluminium, silica, granular activated carbon, synthetic plastic materials) placed at the bottom as a carrier material for a self-immobilized microbial biofilm (Fig. 1h). Continuous up-flow of the wastewater provides thorough mixing of the suspended solids and mass transfer of organics to the biofilm. The AFBRs are widely used in different environmental fields like bioenergy production, domestic wastewater treatment, biodegradation of recalcitrant organic compounds, reduction/oxidation of organic or inorganic contaminants and bioprecipitation of various inorganic compounds *via* oxidation/reduction. They are a prosperous alternative to prevent clogging issues due to fluid up-flow movement and high relative velocity (*1*). The application of the AFBRs to wastewater treatment has advantages over other sludge bioreactors. The adhesion of microorganisms over solid particles with large surface areas enables high biomass concentration and high reaction rates requiring lower bioreactor volume and therefore lower investment costs. Immobilized biomass increases process stability and toxic shock load resistance. Amongst all anaerobic bioreactors treating wastewaters, this model tolerates the highest OLRs (*91*).

Toldrá *et al.* (*92*) studied the effects of temperature and HRT on the treatment of dairy wastewater. They reported that reducing temperature decreased COD removal efficiency from 25 to 10% and the bioreactor performance gradually increased with the increase of HRT. Borja and Banks (*93*) treated ice-cream wastewater in the AFBR. By changing pH, temperature and OLR, they monitored the AFBR performance. Their results showed that the reduction in pH decreased the removal of suspended solids, COD and biogas production, while an increase of OLR shortened the start-up period. The AFBRs can be combined with membrane processes as an alternative solution to some environmental problems. In addition, the AFBRs can be also used to obtain different valuable compounds from wastewaters, such as heavy metals, using sulphate-reducing processes (*94*).

### Horizontal-flow anaerobic immobilized biomass bioreactor

In the horizontal-flow anaerobic immobilized biomass (HAIB) bioreactor microbial biomass is immobilized on different supporting materials (Fig. 1i). The type of support material influences the development of the biofilm, formation strength and mechanical stability (*95*). Moreover, biomass immobilization has a positive influence on cellular retention time, high biomass concentration and microbial diversity (*96*). The best results were obtained when polyurethane foam was used as a supporting material compared to vegetal coal, plastic rings (polypropylene, polyethylene and PET), plastic plates and ceramic matrix (*97*, *98*). Thanks to the high surface area and macroporous structure, polyurethane foam allows forming a gradient of substrate concentration. The HAIB bioreactor has been used mostly due to its low price and good mechanical resistance. The plug-flow regime dominates through the entire length of the bioreactor causing a gradual substrate degradation and growth of a different population of microorganisms along the whole bioreactor length (*99*).

The immobilization procedure of biomass is of great importance. Not properly packed biomass can be washed or cause accumulation of extracellular polymeric compounds and solids from the influent, with bioreactor clogging and pressure drop as a consequence (*100*). The HAIB bioreactor application has been associated with domestic sewage, industrial wastewater, paper industry effluent treatment (*99*), and toxic substances like xylenes, formaldehyde, phenol, pentachlorophenol, toluene, benzene and ethylbenzene removal (*97*, *101*). Souza *et al.* (*102*) have tested the HAIB bioreactor for bioremediation of groundwater contaminated with gasoline and Chatila *et al.* (*103*) have used the HAIB bioreactor for sulfamethoxazole and ciprofloxacin elimination, achieving a removal efficiency of 97-100%.

### Anaerobic fixed-structure bed bioreactor

The anaerobic fixed-structure bed (AFSB) bioreactor as a novel, upgraded version of the HAIB bioreactor (randomly packed-bed) has a lower energy input and sensitivity to environmental variations (pH, temperature and OLR), high sludge retention time and higher substrate conversion rates (*100*). The basic parameter that differs fixed-structure bed bioreactors from expanded- and fluidized-bed bioreactors is the bed porosity. Even though fixed-bed bioreactors have higher bed porosity, they do not need additional energy to maintain expansion or fluidity of the bed, which expanded- and fluidized-bed bioreactors require, and accordingly, they are more efficient (*100*). Higher biomass concentration can be achieved without increasing the solids of the effluent. Since the biomass is fixed throughout the entire length of the bioreactor, sludge dissipation is excluded (*104*).

The AFSB bioreactor has primarily been used for the treatment of sugar cane vinasse (*104*), brewery wastewater (*105*), wastewater containing sulphate (*106*) or wastewater from ethanol production due to its high organic and nutritional content (*107*). The first two-phase anaerobic digestion system for enlarged organic material removal and biogas production from sugar cane vinasse combines acidogenic and methanogenic bioprocess phases. The acidogenic phase is conducted in the AFSB bioreactor and methanogenic phase in the UASB bioreactor. The results pointed out the feasibility of AFSB bioreactor with an overall COD removal higher than 80%. This bioreactor is also characterized by the stable long-term operation (240 days), even at high OLR values (30 kg/m^3^/day). The use of similar bioprocess conditions to the UASB bioreactor resulted in the accumulation of acids with every increase of OLR. Under these conditions, the UASB performance is significantly decreased. Therefore, in the UASB bioreactor methanogenesis conditions have to be established in order to enlarge the efficiency of organic matter degradation and bioenergy recovery.

### Anaerobic membrane bioreactors

Anaerobic membrane bioreactors (AnMBRs) use highly permeable polymeric membranes to retain active biomass, forming a selective barrier and allowing certain components to pass while retaining others. There are three primary AnMBR configurations: external side-stream membrane (Fig. 1j), submerged membrane and submerged membrane with external membrane tank. The submerged anaerobic membrane bioreactor (SAnMBR) is more popular than an external side-stream type, requiring less space, lower energy consumption and causing less fouling. Due to microorganism fouling, adsorption of material between pores or on the surface and high biomass concentrations, it can be complicated to operate the membrane system (*108*). The concentration of active biomass is much higher than in the sludge bioreactors (UASB or EGSB) (*109*). The membrane function is to recover cells or products as well as to separate inhibitory compounds. Membranes are subject to the fouling of materials within pores or on the surface causing the reduction of membrane permeation rate. Granular or powdered activated carbon (GAC or PAC) turned out to be very good at decreasing membrane fouling, strengthening adsorption and potentially enhancing biodegradation (*110*). Activated carbon has been extensively investigated for the removal of pollutants such as pharmaceutically active compounds, xenobiotics, residual organic matter, dyes, hormones, bactericides, phenol and phenolic compounds from wastewaters (*111*, *112*). Biogas production and COD removal efficiency of membrane bioreactors can be considerably enlarged compared to the bioreactors with suspended microbial cells (without any retention mechanisms) due to the active microbial biomass retention (*113*).

The AnMBRs generally have high productivity and relatively good toxic resistance. The improved economy of biogas production can be achieved by using micro- or ultrafiltration systems. They are classified into two main groups: conventional and modified AnMBRs. The conventional AnMBRs are CSTR, UASB, EGSB, AFBR and JFAB (jet flow anaerobic bioreactor). Combination of modified AnMBRs with novel technologies can result in the biogas production with the highest quality. These novel technologies are Anammox (anaerobic ammonium oxidation), dynamic membrane, membrane distillation, forward osmosis, membrane sponge, gas-lifting, and vibrating AnMBRs (*65*). Nonetheless, the application of the novel technologies on a commercial scale requires new studies to give solutions for many technological challenges such as reduction of membrane fouling and cost, product inhibition and methane recovery. Summary of the bioreactor advantages and disadvantages is presented in Table 3.

**Table 3:** Comparison of bioreactor configurations (*1*, *17*, *19*, *40*, *46*-*51*, *61*, *65*, *67*, *76*-*79*, *89*, *90*, *99*, *100*)

Bioreactor configuration	Feedstock	Advantages	Disadvantages
Conventional anaerobic bioreactor			
Anaerobic sequencing batch reactor (ASBR)	wastewaters, tannery waste	easy to operate, simply constructed, low input process, low mechanical requirements, cost-effective	small volume, channelling, clogging, poor self-immobilization, poor transfer of the substrate to the microorganisms
Continuous stirred tank reactor (CSTR)	food waste, animal manure, organic industrial wastes, energy crops	complete mixing of waste and microorganisms, applicable for substrates with high TS, easy to operate, low capital and operating costs, better contact of microorganisms with the substrate	long retention time, high mixing energy consumption, difficulties to retain a high microorganism concentration
Two-stage CSTR system (TPAD, PMAD, HPAD)	animal manure, organic food and industrial wastes, energy crops	system of homogeneous bioreactors, applicable for high TS substrates, easy to operate, low operating costs, washout prevention, *in situ* biogas upgrading	considerable retention time, capital costs, feed of high concentrated substrate
Anaerobic plug-flow reactor (APFR)	farm liquid effluent, slurries of animal manure, cattle residues, distillery wastewater, organic fraction of municipal solid waste	simple to build and maintain, efficient in converting the substrate to biogas, stable to operate, high degree of sludge retention, stable reactor performance	no internal agitation, sedimentation of heavier parts and floatation of lighter parts
Bioreactor with sludge retention system			
Anaerobic contact reactor (ACR)	wastewaters of food processing industry, pulp and paper mills, palm oil mill effluent	high concentration of active microbial biomass, rapidly achieved steady-state times due to mixing, short HRT, high effluent quality, less affected by shock loading, favourable pH, limited biomass washout and change in biogas concentration and composition	sensitive to shock loadings, VFA accumulation
Up-flow anaerobic sludge blanket (UASB)	brewery and molasses wastewater	compactness, high loading rates, low sludge production, short HRT and SRT times, low operating costs with high methane production rates	low total solids, long start-up period, significant wash-out of sludge during the initial phase, impure biogas, incomplete or insufficient removal of organic matter, pathogens and nutrients in the final effluent
Expanded granular sludge bed (EGSB)	brewery, starch, commercial laundry, domestic, municipal and pharmaceutical wastewaters, effluents from the textile industry, dyes and toxic compounds	better substrate-biomass contact, higher throughput, improved internal mixing without dead zones, higher permeability, lower footprint, low operating costs, compact design, removal efficiency up to 90 %, completely closed system with zero emission of odours	long start-up times, problems with biomass retention, granule disintegration, wash-out of hollow granules, the appearance of fluffy granules
Up-flow anaerobic solid-state (UASS)	corn silage, barley straw, wheat straw, organic solid waste	higher processing efficiency, higher volume loading rate, lower investment costs, simple operation and management	utility, scalability, operability and stability are hardly known, the system is limited by its structure, small volume
Anaerobic baffled reactors (ABR)	paper mill effluent, food waste	achieving good COD and solids removal, low sludge production, small footprint	frequent loss of microorganisms from the system, slow growth rates of methanogenesis, long start-up process, sludge washout at high hydraulic stresses, organic loading shock in the initial compartments
Internal circulation (IC)	wastewater from breweries, pulp and paper industry, distilleries, fermentation and petrochemical processes, wastewater from citric acid production	high OLR, effective stress resistance, economic space utilization, excellent operation stability, better treatment performance and faster start-up	high ammonia nitrogen content and presence of toxic substances due to high OLR, unsatisfactory COD removal efficiency, accumulation of VFAs, poor sludge retention, insufficient stability of system
Anaerobic fluidized bed reactor (AFBR) ice-cream, simulated milk, dairy, synthetic dairy wastewaters compact bioreactor size due to short hydraulic retention time, long biomass retention on the carrier, high conversion rates due to fully mixed conditions, high mass transfer rates, no channelling of flow, high organics load size limitations due to the height-to-diameter ratio, high-energy requirements due to high recycle ratios, long start-up period for biofilm formation Horizontal-flow anaerobic immobilized biomass (HAIB) domestic sewage, industrial wastewater, paper industry effluent, toxic substances (phenol, benzene, toluene, ethylbenzene, xylenes, formaldehyde, pentachlorophenol) low price, good mechanical resistance, gradual substrate degradation and microorganism growth, long cellular retention times, high biomass concentrations randomly packed-bed, channelling within the bioreactor, pressure drops Anaerobic fixed-structure bed (AFSB) sugar cane vinasse, brewery wastewater, wastewater containing sulphate, wastewater from ethanol production energy input, high sludge retention time, higher substrate conversion rates sensitivity to environmental conditions, laboratory scale only Anaerobic membrane bioreactor Conventional AnMBRs pharmaceutically active compounds, xenobiotics, residual organic matter, dyes, hormones, bactericides, municipal and domestic wastewater higher concentrations of active biomass, high OLR and HRT, short retention time, good substrate-sludge contact, sufficient mixing and compact design, lower capital costs, tolerance to toxic compounds methane recovery, product inhibition, rapid membrane fouling, membrane cost, low membrane flux, two-stage systems are often required for effective biogas production Modified AnMBRs industrial wastewaters, phenolic compounds from wastewaters, wastewater containing lipids and toxic compounds reduced membrane fouling, enhanced sludge filterability, fewer energy expenses, high nitrogen removal, overcome long start-up period, high OLR, reduced fouling strong shear stress, biogas escape from the external membrane unit, uneconomical for large-scale applications, further studies required to determine the optimal conditions						

## BIOGAS PURIFICATION AND UPGRADING TECHNOLOGIES

The produced crude biogas typically needs to be purified and upgraded for further use. Besides CH_4_ (*φ*=50-70%), biogas contains CO_2_ (*φ*=25-50%), H_2_S (0-5000 mg/L), NH_3_ (0-500 mg/L), N_2_ (*φ*=0-5%), H_2_ (*φ*<3%), O_2_ (*φ*<1%), volatile organic contaminants (<4500 mg/m^3^), siloxanes (<50 mg/m^3^), halocarbons (<200 mg/L) and water vapour (*φ*<5%), all considered as contaminants (*3*). These compounds have to be removed prior to biogas usage either in a heat/energy production or as fuel (biomethane). The presence of CO_2_ and N_2_ lowers the Wobbe index of biogas, which is by definition a quality of combustible gas that allows the air/fuel requirement to be determined (*114*). Unlike natural gas (35.8 MJ/m^3^), the calorific value of biogas (21.5 MJ/m^3^) is smaller due to the large volume of non-combustible CO_2_ that increases the compression and transportation expenses, limiting the economic utility of biogas for generating power directly at the production site (*115*). The O_2_ and H_2_S are also considered as biogas impurities and the combination of these two forms H_2_SO_4_, which can corrode pipelines, gas storage tanks, compressors and engines. Only if these impurities were removed, the purified biogas would have high-quality CH_4_ and could be used for heat and electricity (*115*). The use of biogas as fuel or as an alternative to natural gas requires a stricter purification. Thus, biogas must contain CH_4_ volume fractions higher than 80-96%, *φ*(CO_2_)<2-3%, *φ*(O_2_)<0.2-1%, *γ*(H_2_S)<5-15 mg/m^3^, *γ*(NH_3_)<3-20 mg/m^3^, and *γ*(methylsiloxane)<5-10 mg/m^3^ (*116*). Purified biogas not only helps in reduction in greenhouse gas emissions but also emits fewer hydrocarbons, NO and CO than gasoline or diesel (*117*).

Methods for biogas purification are mainly based on the following mechanisms: absorption, adsorption and membrane-based separation. Absorption is one of the most extensively implemented technologies for CO_2_ and H_2_S separation. It is based on the transfer of CO_2_/H_2_S from the biogas to a liquid scrubbing solution, which can be water, an organic solvent or a chemical solution. Separation principle requires CO_2_ to be more soluble in the scrubber than CH_4_. It can be carried out in single-pass or multi-stage absorption columns. Adsorption is a process in which adsorbate travels from a gas or liquid phase and selectively binds to the surface of a microporous solid phase. The process can be reversed by decreasing or increasing pressure. Membrane separation works on a principle of the difference in chemical affinity and particle size of different molecules. The choice of appropriate technology is related to the specific biogas requirements, local circumstances, it is site-specific and case-sensitive. In the next section, the main physical, chemical, biological and current novel technologies applicable on industrial scale, with a special focus on the removal of CO_2_, H_2_S, O_2_, N_2_, siloxanes, and halocarbons, are described.

### CO_2_ and H_2_S removal

#### Physical upgrading technologies

When using the water scrubbing absorption, the higher aqueous solubility of CO_2_ than of CH_4_ allows selective removal of CO_2_ and H_2_S using water as absorbent. Removal of H_2_S prior to CO_2_ is required because if dissolved in water, it can cause corrosion. The solubility of CO_2_ in the water at 25 °C is nearly 26 times higher than of CH_4_. The biogas is pressurized (0.9-1.2 MPa, 40 °C) and injected from the bottom side of the absorption column, while the water is added from the top flowing towards the counter-current flow of the gas. With the higher pressure, the difference in solubility becomes larger and the CO_2_ and H_2_S are absorbed faster. It should be noted that the huge amount of fresh water is used in the water scrubbing process (*118*).

For organic solvent scrubbing, a mixture of methanol, dimethyl ether and polyethylene glycol can be used as adsorbent, with the higher affinity for CO_2_ and H_2_S than water. Before CO_2_ absorption, it is necessary to separate H_2_S due to the following facts: H_2_S reduces CO_2_ adsorption capacity and its regeneration for the solvent is very demanding. Commercial chemical products Selexol® and Genosorb® are able to absorb three times more CO_2_ than water, meaning fewer liquid inputs in the system and smaller dimensions of the upgrading unit are required. Despite that, the organic solvents need to be regenerated either by depressurizing and/or heating, which is energy-consuming (*119*). The use of the above-mentioned technology is related to the final CH_4_ volume fraction of 98% in purified biogas (*120*).

In the pressure swing adsorption (PSA), CO_2_ and H_2_S can be selectively transferred to a solid surface. PSA separates different gasses from the biogas based on the adsorbent affinity for these gases and their chemical characteristics. The PSA adsorbents must have a large surface area, must be non-hazardous, readily available, stable under long-term operation and selective to CO_2_ and H_2_S molecules. Typically used adsorbents are activated alumina and carbon, carbon molecular sieves, zeolites (zeolite 13, zeolite 5A), polymeric sorbents but also some innovative materials like silicate, metal-organic framework, and silicoaluminophosphate sorbents (*121*). The main characteristics of the PSA process are low energy costs, safety, flexibility of design, equipment compactness and high efficiency (*122*). The PSA system consists of 4 phases: adsorption, blow-down, purge and pressurization that take place in four connected columns running in parallel. The compressed biogas (0.4-1.0 MPa) is injected into the first column where the PSA adsorbent would selectively retain CO_2_ and H_2_S, while the CH_4_ is collected from the top of the column by decreasing the pressure. Once the adsorbent in the first column is saturated, the biogas stream continues to the next column. The regeneration of saturated adsorbent material is performed by decreasing the pressure and releasing the mixture of gasses that contain notable amounts of CH_4_, and therefore, it has to be recycled (*123*). The raw biogas can be purified up to 96-98% of CH_4_ with less than 4% CH_4_ loss within the off-gas stream (*120*).

Membrane separation works on the principles of selective permeability of the membrane material, chemical affinity and particle size. The process can be conducted either as dry (gas/gas separation) or wet (gas/liquid separation). The system efficiency depends on the selection of the membrane and membrane material. Three types of the membrane for biogas upgrading are commercially available: inorganic, polymeric and mixed matrix.

Inorganic membranes are assembled in dense and porous phase. To construct the dense phase, calcium titanate, sliver, palladium, zirconia and nickel are used, while silica, zeolite, alumina and carbon are used for porous phase. Even though inorganic membranes have high thermal and chemical stability, high manufacturing costs limit their application at commercial scale (*124*).

The organic membranes are mostly made of cellulose acetate, polycarbonate, polyesters, polysulfone, polyimide, polyetherimide and polypyrolones. In this type of membranes, the diffusion coefficient and the CO_2_ solubility are higher than in the inorganic ones, resulting in higher permeability. The gas rich in CH_4_ would stay on the side of the membrane with the higher pressure, while the CO_2_ and H_2_S would diffuse to the side with the lower pressure. The mixed matrix membranes (MMMs) are constructed by mixing inorganic filler and organic polymer matrix to achieve higher permeability and selectivity. The combination of these two materials changes the membrane permeability by acting as molecular sieve barrier and by disrupting the matrix structure (*125*). The large permeability difference between CH_4_ and CO_2_/H_2_S (in order to minimize the CH_4_ losses) and efficient biogas purification have to be characteristics of ideal membrane. Today, membrane technology research is focused on the construction of techno-economically feasible polymer with better permeability and without diminishing the selectivity of the membrane.

### Chemical upgrading technologies

Improved scrubbing is obtained by the use of chemical scrubbing where the CO_2_ and H_2_S react chemically with the solvent, resulting in higher absorption capacities and process operation (*126*). Therefore, it is possible to use more compact units, lower liquid recycling rates, and process operation at low absorption and stripping pressures (0.1-0.3 MPa). Chemicals such as alkanolamines (monoethanolamine (MEA), diglycolamines (DGA), diethanolamine (DEA), activated methyldiethanolamine (aMDEA)), bases and some salts (KOH, K_2_CO_3_, NaOH, Fe(OH)_3_ and FeCl_3_) are often used in chemical scrubbing (*123*, *127*). Due to the high selectivity of the chemical solvents towards CO_2_ and H_2_S, the final CH_4_ content of output gas can reach 99% purity with the CH_4_ loss lower than 0.1% (*128*). The energy needed for solvent regeneration, the initial cost of the alkanolamines and their evaporation losses, and solvent toxicity are the main disadvantages of chemical scrubbing.

### Biological upgrading technologies

The biological upgrading technologies are performed under mild process conditions (atmospheric pressure, moderate temperature) contributing to sustainable bio-based production. Hydrogenotrophic CO_2_ removal, also known as biological methanation of CO_2_, is based on the utilization of H_2_ by hydrogenotrophic members of the domain *Archaea* to convert CO_2_ into CH_4_. The hydrogenotrophic CO_2_ removal is an option to chemically store energy as CH_4_, by H_2_ production and the biogas upgrading (*129*). This concept is named power-to-gas (P2G) and except for biogas purification, it can be applied to join solar/wind energy technology with biogas production for the conversion of CO_2_, CO and H_2_ to CH_4_ (*130*).

The hydrogenotrophic CO_2_ removal can be performed *in situ* and *ex situ*. In the *in situ* upgrading, H_2_ is directly injected in a bioreactor where it couples with the CO_2_ which is then converted into CH_4_ (*131*). This conversion leads to a decrease of H^+^ ions and HCO_3_^–^ consumption, causing higher pH values (pH>8.5) and the inhibition of the methanogens. To overcome pH increase, a co-digestion with acidic waste or installation of a pH control device can be a possible solution (*132*). High H_2_ levels contribute to the accumulation of the VFAs and alcohols, which leads to the inhibition of the AD process (*133*). On the contrary, a recent study showed that the pulse H_2_ injections resulted in an increase of H_2_ uptake rates and consequently accumulation of acetate responsible for the induction of homoacetogenic bacteria (*134*). Because the hydrogen solubility in water and hydrogen gas-liquid mass transfer coefficients are low, restricting the bioconversion of CO_2_ to CH_4_, special attention should be paid to the material and type of the module that is used to inject H_2_, recirculation flow, stirring speed and the bioreactor design (*131*, *135*).

The *ex situ* upgrading concept supplies the CO_2_ and H_2_ from external sources resulting in subsequent conversion to CH_4_. The advantages of *ex situ* upgrading concept are as follows: (*i*) because of the separated upgrading, the stability of the conventional biogas process is improved, (*ii*) simpler biochemical process since there is no degradation of organic substrate, (*iii*) biomass-independent process, (*iv*) another external source of waste CO_2_ can be used to improve the process flexibility, and (*v*) suitable to supply power to the rural areas independent from the centralized grid (*131*).

The photosynthetic CO_2_ and H_2_S removal work on the principle of simultaneous bioconversion of CO_2_ and H_2_S through oxygenic photosynthesis carried out by microalgae. The bioconversion can take place either in open or closed photobioreactors. The most extensively studied microalgal species used for photosynthetic CO_2_ and H_2_S removal are *Arthospira, Chlorella, Scenedesmus* and *Spirulina*. These species are recognized by their tolerance to high CO_2_ volume fractions (up to 40-60%) and pH values (9-10) (*136*, *137*). Parameters such as light irradiance (1200-2500 µmol/(m^2^·s), temperature (15-30 °C), pH (7.0-8.0) and dissolved oxygen (<25 mg/L) define the rates of CO_2_ and H_2_S biological fixation by microalgae (*138*). The CO_2_, water, nutrients and mineral salts are transformed into energy components and O_2_ enclosed in the microalgal biomass, while the H_2_S is completely oxidized to sulfate. Microalgal biomass can be used for the extraction of valuable products, biofertilizer production or as a feedstock for biogas production (*139*, *140*). A cost-efficient and simultaneous elimination of CO_2_ and H_2_S, together with a conversion of CO_2_ into microalgal biomass, can be supported by biological upgrading technologies. Still, the major process limitation is the gas-liquid mass transfer of CO_2_ and H_2_. It should be pointed out that the most biological CO_2_ and H_2_S removal technologies are still at a pilot scale, which limits both investment and operating cost data.

### Novel technologies

Novel technologies such as cryogenic separation, *in situ* upgrading and hybrid technologies, are improvements in biogas purification and upgrading. They are cost-effective compared to other mentioned techniques. Still, they are convenient only for the small-scale biogas production that does not require high CH_4_ purity. Therefore, the knowledge gap between pilot tests and large-scale operations has to be bridged.

Cryogenic separation is a technology in biogas purification that works on a principle of the different condensing temperatures of different gases. The raw biogas is gradually cooled (~-170 °C) and compressed (8 MPa) through a series of compressors and heat exchangers, which remove liquid CO_2_, the remaining CO_2_ in the solid phase, siloxanes and halogens (*141*). However, removing H_2_S and water prior to cryogenic separation is necessary to avoid freezing. The advantages of this process are high CH_4_ purity (99%), no chemicals required, quite low CH_4_ loss (<0.1%), upgraded biogas is at high pressure and therefore ready to use as a vehicle fuel. Also, pure CO_2_ is obtained as a by-product. Nevertheless, it is still energy-intensive technology with the high investment and operation costs and only a few plants under operation at a global scale (*120*, *136*).

*In situ* upgradation technology works on the principle of using the desorption process by applying moderate recirculation rates. In the first stage, sludge circulates through the desorption column and recirculates back to the bioreactor. In the desorption column, liquid sludge undergoes counter flow of O_2_ and N_2_ by which CO_2_ dissolved in the sludge is desorbed (*142*). The airflow rate in the desorption column was found to be a key variable. The increase of air flow rate in the desorption column is related to the lower CO_2_ and H_2_S content in the upgraded biogas, but CH_4_ losses are also increased (*143*). The pH increases up to pH=8.0 lead to inhibition of methanogenesis and high CH_4_ loss in the system. Although this concept was first presented 20 years ago, up to this time, it has been in its initial phase and only tested at pilot scale (*144*).

### Hybrid technologies

One promising way to overcome the disadvantages of the above-mentioned technologies is to combine them by creating hybrid technologies. Combining membrane separation with conventional processes such as water scrubbing absorption, chemical scrubbing or cryogenic separation exceeds the conventional processes in terms of low operational costs, high CO_2_ and H_2_S-capture efficiency, higher yields of CH_4_, competitiveness and less energy consumption (*145*, *146*). Another hybridized process is an industrial lung in which carbonic anhydrase (CA) enzyme catalyzes the conversion of [REMOVED HYPERLINK FIELD]CO_2_ and H_2_CO_3_ that are removed in an absorber column. Small scale experiments demonstrated that it can purify biogas up to 98% of CH_4_ with a CO_2_ less than 1%, the industrial lung process is limited by short enzyme lifetime and high enzyme production costs (*147*). The hybrid technology combines temperature-membrane-cryogenic process characterized by lower energy consumption than individual conventional technologies (*146*). Hybrid technologies have to be more investigated in order to merge the advantages of two or more upgrading technologies and to improve the biogas purification.

#### O_2_ and N_2_ removal

The O_2_ and N_2_ are not produced by AD but they are found at high volume fractions in landfill gas when biogas is collected by vacuum generation as a consequence of air infiltration. The relevant technologies for both O_2_ and N_2_ elimination are pressure swing adsorption (PSA), membrane and cryogenic separation. The advantages of the PSA and membrane technology are removing O_2_ and N_2_ along with CO_2_, low energy demand, and low level of greenhouse gas (GHG) emission. They are easy to handle and maintain. Nevertheless, before the PSA, H_2_S and water have to be removed. Cryogenic separation produces CO_2_ as by-product and can remove different impurities. The separation of O_2_ and N_2_ from the rest of biogas is based on the temperature difference in the condensation of biogas compounds, it requires great energy consumption, investment and operating costs (*123*).

#### Siloxane, volatile organic compound and halocarbon removal

Siloxanes are polymeric organic silicones usually used in cleaning products and cosmetics. The presence of siloxanes and formation of silicon oxide sediments can cause malfunctioning of engines and valves, overheating and abrasion (*148*). The siloxane removal technology is based on the adsorption on activated carbon or silica gel and cryogenic separation. The ability to remove siloxanes is poor as a consequence of the strong mass transfer limitations mediated by their extremely low aqueous solubility (*149*). Volatile organic compounds (toluene, VFAs) and halocarbons are removed by activated carbon adsorption in two parallel packed bed columns (*136*). The microorganisms from the genus *Pseudomonas* are able to degrade hexamethylcyclotrisiloxane and octamethylcyclotetrasiloxane (*149*). However, the biological removal of siloxanes, volatile organic compounds and halocarbons is still not enough explored and applied. Summarized overview of upgrading technologies is given in Table 4.

**Table 4:** Overview of upgrading technologies for biogas purification (*115*, *118*, *120*, *122*, *125*, *128*, *130*, *136*, *142*, *146*)

Upgrading technology		*φ*(CH_4_)/%	*φ*(CO_2_)/%	*γ*(H_2_S)/(mg/L)			*φ*(CH_4_)_loss_/%	*E*/(kWh/Nm^3^)		Advantages	Disadvantages	
Physical												
Water scrubbing absorption		95-98	<2	<2			<2	0.20-0.50	high efficiency, simultaneous removal of H_2_S, low CH_4_ losses, tolerance to impurities, possible regeneration, simple operation			expensive investment and operation, clogging due to bacterial growth, requires huge amount of fresh water
Organic solvent scrubbing		93-98	<2	<1			<4	0.10-0.33	economical, simultaneous removal of organic components, H_2_S, NH_3_, HCN and H_2_O, energetically more favourable than washing with water, regeneration with low-temperature waste heat			expensive investment and operation, difficult operation, insufficient operation when stripping/vacuum applied, reduced operation by glycol dilution with water
Pressure swing adsorption (PSA)		>96-98	1-2	2			<4	0.16-0.43	low energy used, no chemicals required, no water demand, high pressure but regenerative, no microbial contamination and impurities			H_2_S pretreatment required, expensive investment and operation, complex setup
Membrane separation		90-99	1–3	2			<5	0.18-0.35	H_2_S and H_2_O are removed together with CO_2_, simple construction and operation, no chemicals required			unstable over the long term, pretreatment required, multiple steps required (modular system) to reach high purity
Chemical												
Chemical scrubbing		>98	<1	<4			<0.1	0.05-0.18	high efficiency, cheap operation, regenerative, more CO_2_ dissolved than with water, very low CH_4_ losses			use of chemicals, corrosion, expensive investment, heat required for regeneration, decomposition and toxicity of the amines or other chemicals
Biological												
Hydrogenotrophic removal		98	7.8	38			<1		mild process conditions, enhancement of CH_4_, no unwanted end products, low operating costs			still on an experimental basis, tested only on a small scale, further developments to increase the H_2_ gas-liquid transfer
Photosynthetic removal	97-99		10	0-0.5		<1		0.05-0.10	mild process conditions, tolerance to high CO_2_ concentrations and pH values, extraction of high value-added products			poor gas-liquid mass transfer of CO_2_ and H_2_, pilot scale, limitation on investment and operating cost data
Novel												
Cryogenic separation	99		<2	<1	<0.1			0.42-1	high purity of CO_2_ and CH_4_, no chemicals required, upgraded biogas at high pressure, no further compression is required, low extra energy cost to reach liquid biomethane			high capital and operating costs, high energy required for equipment such as compressors and heat exchangers, pretreatment required, removing H_2_S and H_2_O prior to cryogenic separation
*In situ* upgradation technology	95				<2				cost-effective, easy to operate			high CH_4_ loss, appropriate only for a small scale, limited by gas-liquid mass transfer
Hybrid technologies	95-98		<1						low operating costs, high CO_2_ and H_2_S-capture efficiency, higher yields of pure CH_4_, competitiveness and less energy consumption			small scale production, limited by enzyme lifetime, high enzyme production costs

*E*=energy consumption (kWh/Nm^3^), Nm^3^=normal cubic metre of biogas at standard conditions (*T*=273.15 K and *p*=101 325 Pa)

## DIGESTATE UTILIZATION

During the AD, together with biogas, a digested substrate called digestate is produced. The digestate is an abundant source of nutrients such as nitrogen, phosphorus, potassium, micronutrients and organic matter, which is applied to soils as agricultural biofertilizer (*150*). Digestate is a high-quality and preferable fertilizer to raw animal manure because of the better homogeneity and nutrient availability, lower C/N ratio, reduced odours and pathogens, and lower risk of soil contamination (*151*). Leaving the bioreactor, digestate is not fully stabilized as it contains pathogens and drains off heavy metals. Before its use on the soil, it needs proper handling and special treatment (*150*).

Digestate can be separated in semi-solid (10-30% dry matter) and liquid (5-15% dry matter) fraction. To reduce the costs (storage, transportation and application) a number of techniques are developed to upgrade the liquid digestate: dewatering, composting, drying, granulating, pelletizing, precipitation, filtration, steam evaporation, membrane separation and many others. The choice of upgrading technology depends on market demands and the location of the plant. The quality and stability of the solid fraction can be improved through composting (*152*). Dried or composed digestate is physically more stable with fewer emissions to air when kept in open storage. The digestate from the AD process can also be co-composted with fresh organic waste or applied as inoculant instead of commercial microbial cultures, which is economically more favourable (*153*). More applications of the solid and liquid fraction of various digestates have been shown in Table 5 (*2*, *153*-*163*).

**Table 5:** The application and advantages of solid and liquid fraction of digestate

Source of digestate	Fraction	Application and advantages		Reference	
brown macroalgae	solid	phenol production		(*154*)	
cattle slurry mixed with energy crops (maize silage and triticale silage)	liquid	biofertilizer		(*155*)	
food waste	liquid	production of biochar by pyrolysis		(*156*)	
maize silage and chicken manure	liquid	plant fertilizer and soil conditioner		(*157*)	
mixture of cow manure, cheese whey, poultry manure, olive pomace and corn silage	liquid	production of an enzyme (exo- and endoglucanase, xylanase, β-glycosidase and laccase)		(*158*)	
mixture of manure, slurry, corn silage and sugar beet pulp	solid	production of pellets and briquettes		(*159*)	
mixture of pig slurry, olive pomace, maize silage, sorghum silage and onion scraps	solid	bio-oil production		(*150*)	
mixture of urban secondary effluent wastewater and digestate	liquid	growth and selection of microalgae (*Chlorella* and *Stigeoclonium*) and cyanobacteria (*Oscillatoria* sp., *Aphanocapsa* sp. and *Chroococcus* sp.)		(*160*)	
municipal organic waste	solid	organic supplement		(*161*)	
pig slurry	liquid	recycled as a solvent to dilute the raw material (silage maize) going into the system		(*2*)	
sewage sludge and source-segregated biodegradable waste	liquid	nitrogen removal of old landfill leachate		(*162*)	
swine manure and maize	liquid	significant reduction of the toxicity, high removal efficiency of ammonia, total nitrogen and phosphate		(*163*)	
wood chips	liquid	improved composting and digestate stability, decreased NH_3_ emission but multiplied N_2_O emission		(*153*)	



## APPLICATION OF MOLECULAR BIOLOGY AND NEXT-GENERATION SEQUENCING TO IMPROVE BIOGAS PRODUCTION

Since the AD is a complex microbial process, a broad range of studies has been recently performed in order to understand the relationship between the microbial community structure, operating conditions and bioprocess performance. Methanogenic archaeal community (or methanogens) has an important role in the last step of anaerobic organic matter degradation, that is, methanogenesis. The detailed understanding of how methanogens interact with other organisms in their environment is still a black box for microbiologists and engineers. The most abundant microbial populations present in the bioreactor can be identified by traditional molecular biology technologies. Recently, the determination of both the most abundant and minor populations can be done by the newly developed sequencing techniques (*164*). These sequencing techniques provide a valuable tool for the understanding of the microbiological systems and their function along with different aspects of anaerobic degradation and biogas process optimization. They are based on the detection and sequencing of DNA molecules extracted directly from microbial cells. Most of them use amplification of the 16S rRNA gene sequence, which has been specified for each microorganism and considered as a gold standard for identification and presence of bacteria and archaea in the environment. The standard choice of methanogen-specific genetic marker is the mcrA gene for methanogenic microbial populations. The most common, rapid and cost-effective techniques used for the precise detection of methanogenic populations are terminal restriction fragment length polymorphism (TRFLP) (*165*), denaturing gradient gel electrophoresis (DGGE) (*164*, *166*), quantitative real-time polymerase chain reaction (qPCR) (*167*, *168*) and ion torrent PGM (personal genome machine) technique (*169*). For a more complete characterization of the microbial community structure, current approach favours metagenomics, also called next-generation sequencing (NGS) technique. Nowadays, several NGS platforms are available and used to improve biogas optimization: 454 pyrosequencing (Qiagen), Illumina MiSeq and HiSeq (Illumina Inc.), SOLiD (Life Technologies), Ion Torrent (Thermo Fisher) and MinION (Oxford Nanopore Technologies) (*76*). Most studies have been focused on exploring the microbial community inside the bioreactors without taking into account the whole biogas production system (including storage and feeding together with the post-digestion systems). By using next-generation sequencing tools, it is possible to get useful information on functional diversity and gene expression at the community level as well as control the entire bioprocess in a more effective way. Future optimizations of biogas production systems have to be based on the combination of different NGS methods for the study of microbial community dynamics and functional activities.

## CONCLUSIONS

The development of complex biorefinery systems based on underutilized and low-value biomass must be in focus of the biogas production. Generating energy and materials from these unconventional bio-resources is a promising solution for the environmental protection. Different biotechnological methods can be applied for high-strength organic waste treatment due to their low energy consumption, less residual sludge generation and efficient energy recuperation. The anaerobic digestion (AD) is a useful method of recovering energy from organic waste while diminishing the environmental impact of the waste. The produced biomethane can be used as a replacement of fossil fuels in the heat and electricity production, substitute for natural gas for domestic and industrial use, used in co-generation or as a vehicle fuel.

Many different bioreactor configurations and operational techniques have been developed for AD. The installation and operation costs, as well as maintenance, are factors that significantly influence the economics of biogas production. The choice of the bioreactor type has to consider nature and strength of the feedstock, construction and operation costs, infrastructural support, climatic conditions, availability and the level of skills of the local employees, and energy cost as well as prospects for disposal of effluent and digestate.

Regarding biogas purification and upgrading technologies, physical and chemical technologies are in general at high technology readiness levels, while biological methods are still new and commercially poorly applicable. Nevertheless, the development of biological methods is rapid and gives new perspectives for integrating different forms of renewable energy. Apart from upgrading, they can offer electricity storage advances and decoupling bioenergy production from biomass availability. These methods are able to support a simultaneous elimination of CO_2_ and H_2_S, with a CO_2_ transformation into microalgal biomass for the production of biofertilizers or highly valuable products. The novel upgrading technologies are a prospective alternative for overcoming the challenges of current upgradation technologies. Since the AD is a complex microbial process that uses novel molecular biology and next-generation sequencing, it would be possible to control and regulate the process in a more effective way. It is necessary to pay attention to the whole biogas production system and to understand the relationship among the microbial community structure, operating conditions, bioprocess performance and the post-digestion step.
